# Abdominal synthetic CT generation for MR-only radiotherapy using structure-conserving loss and transformer-based cycle-GAN

**DOI:** 10.3389/fonc.2024.1478148

**Published:** 2025-01-03

**Authors:** Chanwoong Lee, Young Hun Yoon, Jiwon Sung, Jun Won Kim, Yeona Cho, Jihun Kim, Jaehee Chun, Jin Sung Kim

**Affiliations:** ^1^ Department of Radiation Oncology, Yonsei Cancer Center, Heavy Ion Therapy Research Institute, Yonsei University College of Medicine, Seoul, Republic of Korea; ^2^ Medical Physics and Biomedical Engineering Lab (MPBEL), Yonsei University College of Medicine, Seoul, Republic of Korea; ^3^ Department of Radiation Oncology, Washington University in St. Louis, St Louis, MO, United States; ^4^ Department of Radiation Oncology, Gangnam Severance Hospital, Yonsei University College of Medicine, Seoul, Republic of Korea; ^5^ Oncosoft Inc., Seoul, Republic of Korea

**Keywords:** MR-linac, abdominal synthetic CT, structure consistency loss, transformer, unsupervised learning

## Abstract

**Purpose:**

Recent deep-learning based synthetic computed tomography (sCT) generation using magnetic resonance (MR) images have shown promising results. However, generating sCT for the abdominal region poses challenges due to the patient motion, including respiration and peristalsis. To address these challenges, this study investigated an unsupervised learning approach using a transformer-based cycle-GAN with structure-preserving loss for abdominal cancer patients.

**Method:**

A total of 120 T2 MR images scanned by 1.5 T Unity MR-Linac and their corresponding CT images for abdominal cancer patient were collected. Patient data were aligned using rigid registration. The study employed a cycle-GAN architecture, incorporating the modified Swin-UNETR as a generator. Modality-independent neighborhood descriptor (MIND) loss was used for geometric consistency. Image quality was compared between sCT and planning CT, using metrics including mean absolute error (MAE), peak signal-to-noise ratio (PSNR), structure similarity index measure (SSIM) and Kullback-Leibler (KL) divergence. Dosimetric evaluation was evaluated between sCT and planning CT, using gamma analysis and relative dose volume histogram differences for each organ-at-risks, utilizing treatment plan. A comparison study was conducted between original, Swin-UNETR-only, MIND-only, and proposed cycle-GAN.

**Results:**

The MAE, PSNR, SSIM and KL divergence of original cycle-GAN and proposed method were 86.1 HU, 26.48 dB, 0.828, 0.448 and 79.52 HU, 27.05 dB, 0.845, 0.230, respectively. The MAE and PSNR were statistically significant. The global gamma passing rates of the proposed method at 1%/1 mm, 2%/2 mm, and 3%/3 mm were 86.1 ± 5.9%, 97.1 ± 2.7%, and 98.9 ± 1.0%, respectively.

**Conclusion:**

The proposed method significantly improves image metric of sCT for the abdomen patients than original cycle-GAN. Local gamma analysis was slightly higher for proposed method. This study showed the improvement of sCT using transformer and structure preserving loss even with the complex anatomy of the abdomen.

## 1 Introduction

Magnetic resonance (MR) images are widely used in radiotherapy, which could identify target and organ-at-risks with excellent soft tissue contrast (1). Recent studies reported the potential benefits of MR-guided radiation therapy (MRgRT) than computed tomography (CT) based image-guided radio therapy (2–4). However, lack of electron density information of MR precludes dose calculation, requiring additional CT scans for treatment planning. The use of multimodal imaging could result in geometric error of 2-5mm during the registration process between CT and MR (5–11). This error could result in systematic geometric deviations, leading to potential underdosage or overdosage of the tumor area and thus compromising the effectiveness of tumor control (12). Especially for the abdomen, significant organ motion and frequent changes in intestinal gases, such discrepancies are further amplified, increasing uncertainty throughout the treatment (13). Additionally, the acquisition of MR images from MR-Linac is more susceptible to degradation due to the B0 field inhomogeneity induced by Linac components (14). However, for clinically streamlined MR-only radiotherapy, the use of MR-Linac is necessary for sCT.

MR-only radiotherapy has been proposed in several studies to mitigate geometric discrepancies (15). By eliminating planning CT imaging and relying solely on MR, MR-only radiotherapy reduces uncertainties from the registration process, decreases the workload of medical professionals, and protects patients from additional radiation exposure from additional CT scans (16). However, reconstructing synthetic CT (sCT) images is necessary to obtain the electron density required for treatment dose calculation. Conventional approaches of sCT are bulk density override and atlas-based methods (17). The bulk density override approach divides the MR into several classes, such as air, bone, soft tissue, and fat, assigning a homogeneous electron density to each segment (18). However, this method is time-consuming when performed manually and does not consider tissue heterogeneity (19). The atlas-based method uses co-registered MR-CT in an atlas to obtain the sCT for the desired MRI, but it can lose robustness when the anatomical structure significantly differs from the existing atlas, and due to the numerous registrations required, it can be extremely time-consuming (19, 20).

Recently, since its introduction by Han in 2017 (21), the deep learning method has proven to be much faster and more accurate than the previously mentioned methods. Building on this study, many studies explored sCT generation for head and neck or pelvis (21–26). However, few studies investigated abdominal sCT due to challenges such as organ motion and the presence of air bubbles, which degrade MR images (27). Furthermore, existing studies for synthetic CT generation for MR-Linac systems have predominantly utilized convolutional neural networks (CNNs), which, despite their effectiveness, often fall short in capturing the complex dynamics of abdominal anatomy (28–30). Transformer, initially applied in natural language processing, was introduced to computer vision as the Vision Transformer (ViT) (31, 32). ViT successfully overcame the limitations of CNNs, which were widely used in the medical image field, by capturing strong correlations among global features of an image through the multi-headed self-attention mechanism (31). Transformer-based networks for synthetic CT generation across various modalities were reported superiority than CNN (33–35).

This study introduces a novel approach for generating abdominal sCT for MR-only radiotherapy. First, this method integrates the Shifted Window U-net Transformer (Swin-UNETR) with an unsupervised cycle generative adversarial network (cycle-GAN). Unlike conventional methods that rely solely on CNNs structures, our approach leverages the strengths of both transformers and u-net in capturing detailed anatomical features and global context. Second, we applied a structure-conserving loss to maintain geometric consistency between the MR and sCT images. We employed the modality independent neighborhood descriptor (MIND) loss to extract geometric features that are consistent across different modalities (36). The aim of this study is to assess the feasibility and performance of this hybrid model with structure conserving loss in improving sCT quality and accuracy, particularly in the challenging abdominal region (36–39).

## 2 Materials and Methods

### 2.1 Patient data characteristics

**Table 1** describes the characteristics of the MR and CT images used in this study. We collected 120 abdominal cancer patients who underwent radiotherapy using Elekta Unity between September 2, 2021, and June 1, 2023, including their T2 MR and CT images. The CT images were scanned with the SOMATOM Definition AS (Siemens Healthcare, Erlangen, Germany). The MR images were scanned using the Ingenia 1.5T MR (Philips, Amsterdam, Netherlands) integrated in Unity (Elekta, Stockholm, Sweden). The range of patients age was 31 to 91. The range of volume size for MR images was (480 – 800) × (480 – 800) × (250 – 300), and for CT images was 512 × 512 × (117 – 543).

**Table 1:** Data characteristics of magnetic resonance (MR) and computed tomography (CT) images.

	MR	CT
Number of patients	120	120
System	Ingenia 1.5T	SOMATOM Definition AS
Mean volume size	800 × 800 × 250	512 × 512 × 203
Mean resolution (mm^3^)	1.02 × 1.02 × 2.60	0.57 × 0.57 × 1.19
Sequence	T2 3D Tra 5min	-

### 2.2 Data preprocessing for CT and MR

Deformable registration was performed using Python 3.9 and Simple-ITK. All images were normalized to have a size of 256 × 256 and a resolution of 0.83 × 0.83 mm^2^. The intensity of MR images was normalized using histogram matching. For CT images, the Hounsfield unit (HU) values were clipped to the range from -1024 to 3071 HU. The largest connected component within the CT image were identified, and an algorithm creating a body mask through binary processing was used to remove external structures. After removing external structures from the images, all patients were aligned based on the coordinates of the spine. The datasets were split into 80, 20, 20 for training, validation, and testing, respectively.

### 2.3 Training details of proposed sCT

#### 2.3.1 Baseline architecture

**Figure 1:**
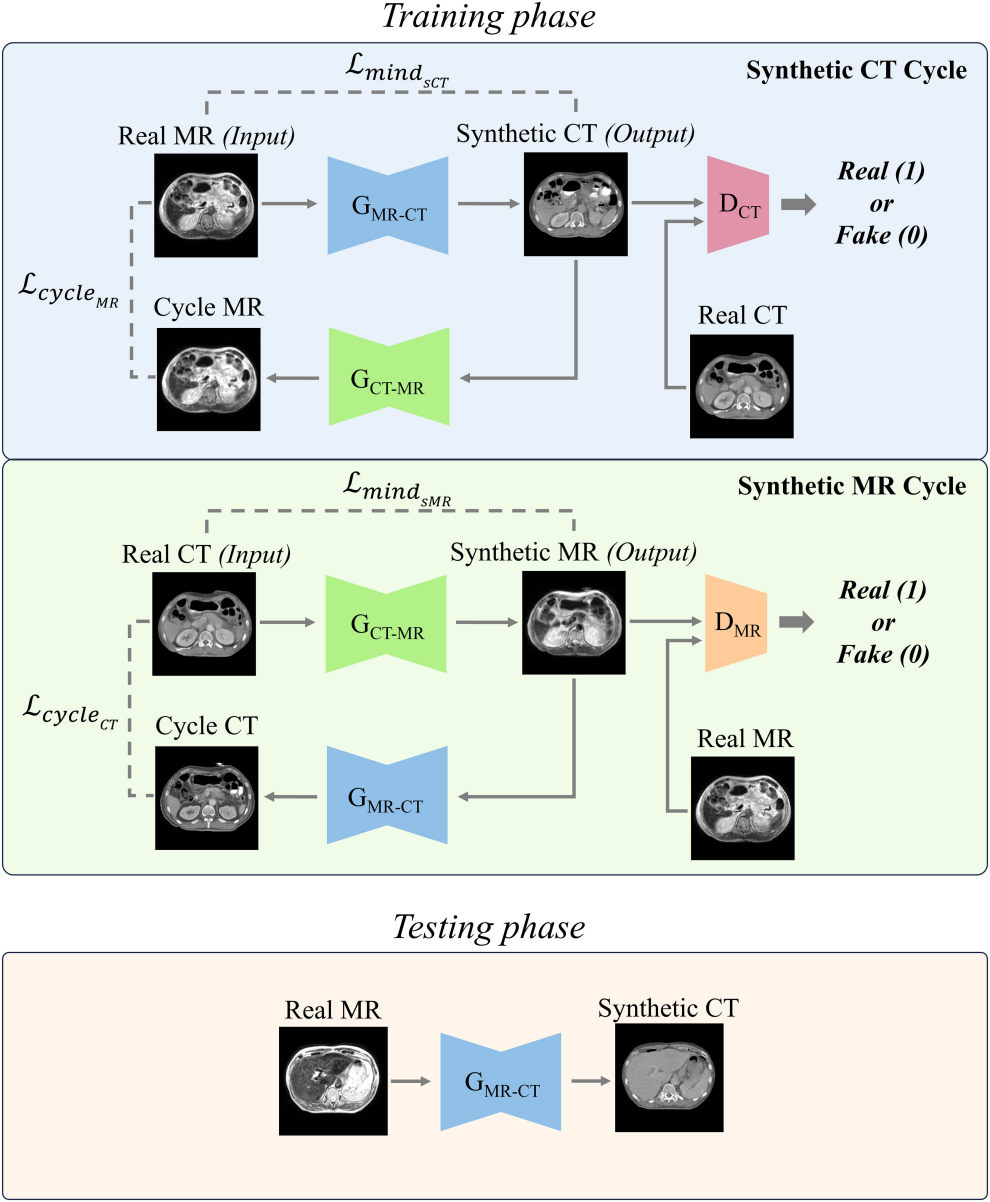
Two distinct cycles include generating synthetic CT (sCT) images and synthetic MR (sMR) images. Each cycle performed a series of transformations between the MR and CT domains to ensure bidirectional synthesis and consistency. In the testing phase, the trained MR-to-CT generator was used to produce synthetic CT images.

The overall architecture is illustrated in **Figure 1**. This study utilized the cycle-GAN (39) comprised of two generators, which produce CT and MR images, respectively. Additionally, there are two Discriminators, which discriminate between generated and planning CT and MR images. Both sets, are trained in a competitive manner. The primary goal of CT generator is to generate sCT images that are indistinguishable from real ones. Conversely, CT discriminator aims to discern whether a given image is genuine or artificially created. The generator and discriminator of MR operate under similar principles. The network hyperparameters for training the generator and discriminator are as follows. The input size for the model was set to 256 × 256 pixels to standardize the resolution of the data. Training was conducted over a total of 100 epochs, using the Adam optimizer with a learning rate of 0.0001. The learning rate was gradually reduced in a linear manner from 30th epochs until it reached zero at the end of final epoch.

#### 2.3.2 Modified generator network of cycle-GAN

**Figure 2:**
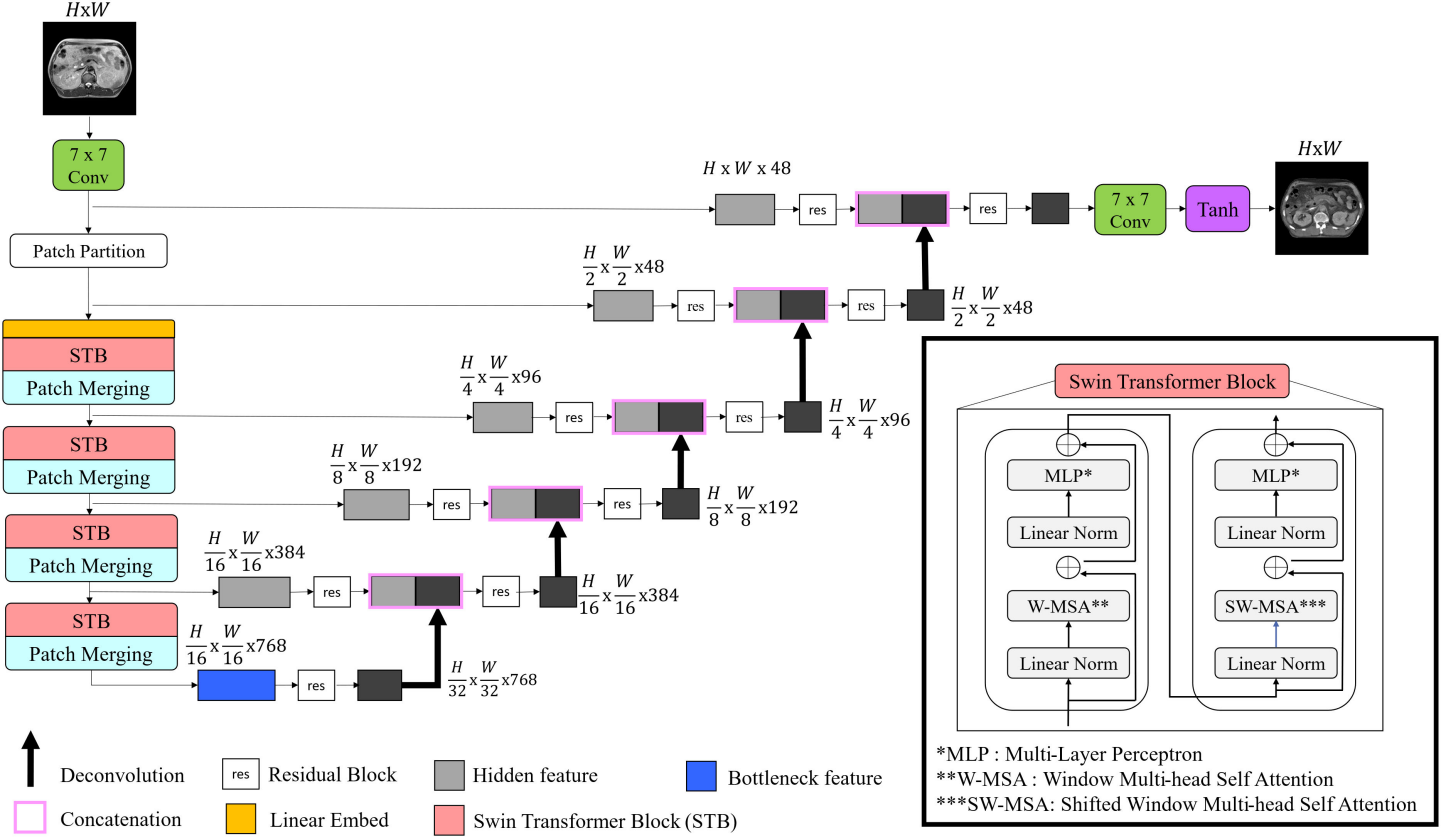
Illustration of proposed generator architecture. The model begins with a convolutional layer for input image processing, followed by Swin transformer blocks that enhance feature extraction. The features are then merged and passed through the encoder-decoder pathway to reconstruct synthetic images.

**Figure 2** depicted architecture of the modified generator used in this study. Recently, Swin-UNETR has gained prominence in medical image segmentation tasks, achieving state-of-the-art results on datasets such as the Medical Decathlon and the Multi-Atlas Labeling Beyond the Cranial Vault segmentation challenge (37). For this study, a modified Swin-UNETR was employed as the primary generator network, tailored for 2D operations. To enhance feature extraction, a 7 × 7 convolutional layer was added both before and after the main Swin-UNETR network.

#### 2.3.3 Loss functions

Cycle-GAN, being unsupervised learning, lacks a direct ground-truth, rendering one-to-one mapping unfeasible. To overcome this limitation, the following loss functions are incorporated as proposed by Zhu et al. (39).

1) The adversarial loss employed is the least square loss. The goal of discriminator is to classify real images as 1 and fake images as 0, whereas the objective of generator is to make discriminator classify the generated images as Equation 1. The loss functions to be minimized for generator and discriminator are as follows:


(1) $$\begin{array}{c} L_{G_{MR-CT}}=\lambda_{G}(\left(D_{CT}\left(G_{MR-CT}\left(MR\right)\right)-1{)}^{2}\right)\\ L_{D_{CT}}=\lambda_{D}\left({\left(D_{CT}(G_{CT}\left(MR\right))\right)}^{2}+{\left(D_{CT}\left(CT\right)-1\right)}^{2}\right)\;+\; \end{array}$$

(2) $$\begin{array}{c} L_{G_{CT-MR}}=\;\lambda_{G}{\left(D_{MR}\left(G_{CT-MR}\left(CT\right)\right)-1\right)}^{2}\\ L_{D_{MR}}=\lambda_{D}\left({\left(D_{MR}(G_{MR}\left(CT\right))\right)}^{2}+{\left(D_{MR}\left(MR\right)-1\right)}^{2}\right) \end{array}$$

$G_{MR-CT},G_{CT-MR},D_{CT},D_{MR}$ refer to CT generator, MR generator, CT discriminator, and MR discriminator, respectively.

2) The definition of cycle consistency loss is to compare the image reconstructed back to the original domain from the synthetic image with the real input image using the L1 loss. The equation is as follows:


(3) $$\begin{array}{c} L_{cycle_{MR}}=\lambda_{cycle}{\left\|MR-G_{CT-MR}\left(G_{MR-CT}\left(MR\right)\right)\right\|}_{1}\\ L_{cycle_{CT}}=\lambda_{cycle}{\left\|CT-G_{MR-CT}\left(G_{CT-MR}\left(CT\right)\right)\right\|}_{1} \end{array}$$

3) Identity loss in cycle-GAN measures how well the generator preserves the original image’s features when transforming images within the same domain. It ensures that when an image is processed by its corresponding generator, the resulting output image remains similar to the input. This similarity is quantified using the L1 loss:


(4) $$\begin{array}{c} L_{idt_{CT}}=\lambda_{idt}{\left\|CT-G_{MR-CT}\left(CT\right))\right\|}_{1}\\ L_{idt_{MR}}=\lambda_{idt}{\left\|MR-G_{CT-MR}\left(MR\right))\right\|}_{1} \end{array}$$

values for $\lambda_{D}$ , $\lambda_{G}$ , $\lambda_{cycle}$ , and $\lambda_{idt}$ were set to 0.5, 1, 10 and 5, respectively.

#### 2.3.4 Structure Conserving Loss function

This task involves performing style transformation while preserving the geometric structure between MR and sCT. In the abdominal region, obtaining paired data is often challenging, and since this study is conducted using unsupervised learning, there is no explicit constraint between MR (input) and sCT (output). To increase the geometric consistency between the input image and the target image, Modality Independent Neighborhood (MIND) loss was applied (36). This method has been shown to improve performance in generating Head and Neck sCT (40). MIND compares local image structures instead of intensity-based comparisons. MIND for an image I is defined as follows (36).


(5) $$MIND\left(I,\;\text{x},\;\text{r}\right)=\frac{1}{n}\exp\left(-\frac{D_{p}\left(I,\;\text{x},\text{ x}+\text{r}\right)}{V\left(I,\text{ x}\right)}\right)$$

(6) $$D_{p}\left(I,\;x,x+r\right)=\sum_{p\in P}{\left(I\left(x+p\right)-I\left(x+r+p\right)\right)}^{2}$$

(7) $$V\left(I,\;\text{x}\right)=\frac{1}{N}\sum_{\text{n}\in N}D_{p}\left(I,\text{x},\text{ x}+\text{n}\right)$$

Here, N is the number of pixels surrounding pixel x, which was set to 8. Dp​ represents the distance between patches, and V is the mean of the distances of the N neighboring patches. Direct computation of Dp​ is computationally expensive; thus, it was implemented using convolution operations as follows.


(8) $$D_{P}\left(I,\;x,\;x+a\right)=C*\left(I-I^{\prime }\left(a\right)\right)$$

**Figure 3:**
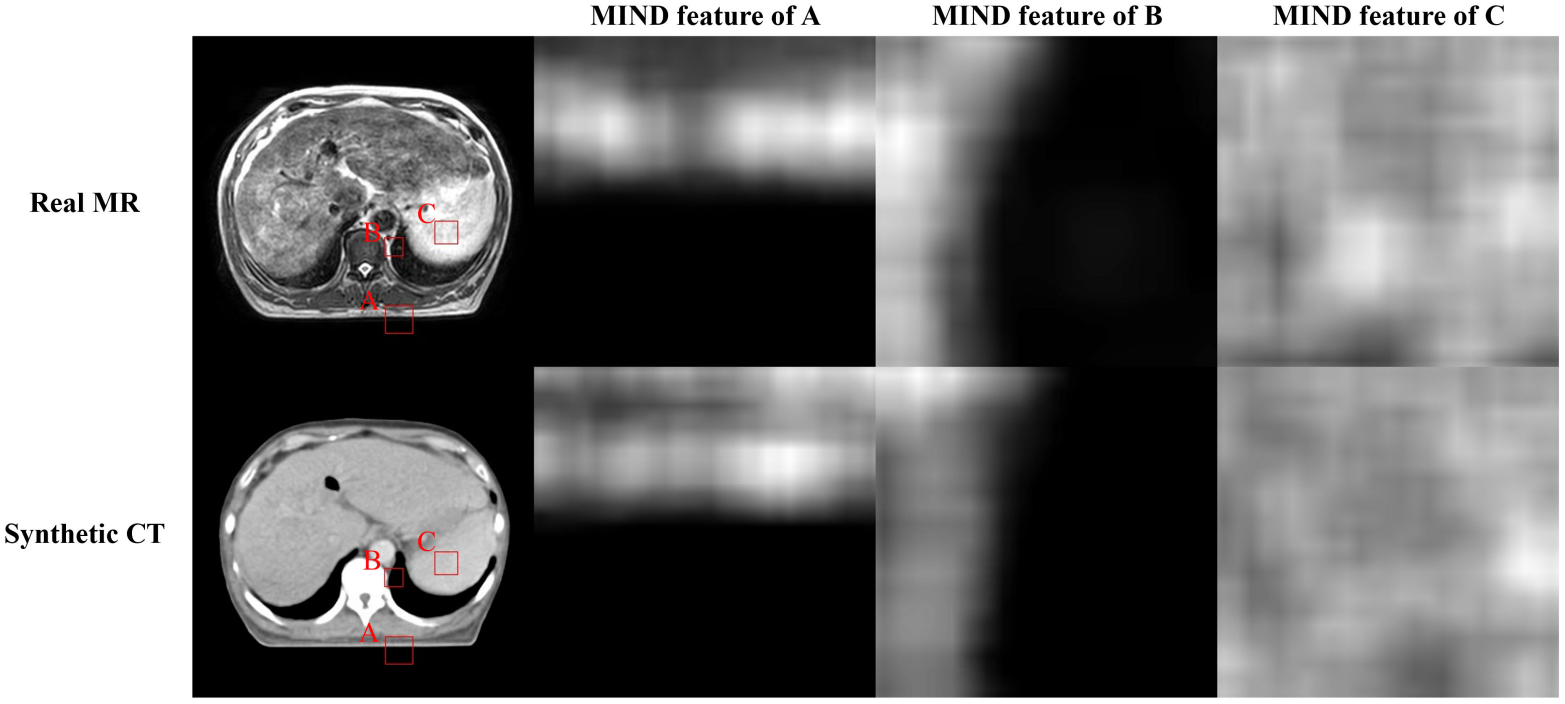
Illustration of the magnetic resonance (MR), synthetic computed tomography (CT), and modality independent neighborhood (MIND) features of them. First row presents real MR image and MIND features in three different positions **(A-C)**. Second row depicts sCT image and MIND features in same positions as MR image.

C is a kernel with all weights set to 1 and the same size as P, and $I^{\prime }\left(a\right)$ is the image I translated by a. This operation simplifies the calculation of the derivative. A visual example of MIND features is depicted in **Figure 3**.

Structure conserving loss that employing MIND is defined as follows:


(9) $$\begin{array}{c} L_{mind_{sCT}}={\left\|MIND\left(MR\right)-MIND\left(G_{MR-CT}\left(MR\right)\right)\right\|}_{1}\\ L_{mind_{sMR}}={\left\|MIND\left(CT\right)-MIND\left(G_{CT-MR}\left(CT\right)\right)\right\|}_{1} \end{array}$$

#### 2.3.5 Total Loss

$G_{MR-CT}$ and $G_{CT-MR}$ were ultimately trained to minimize the follows:


(10) $$\begin{array}{c} L_{sCT}=L_{G_{MR-CT}}+L_{cycle_{MR}\;}+L_{idt_{CT}}+L_{mind_{sCT}}\\ L_{sMR}=L_{G_{CT-MR}}+L_{cycle_{CT}\;}+L_{idt_{MR}}+L_{mind_{sMR}} \end{array}$$

The CT discriminator and MR discriminator were trained to minimize $L_{D_{CT}}$ and $L_{D_{MR}}$ as presented in (1).

### 2.4 Evaluation of proposed method

#### 2.4.1 Image quality

The similarity between the sCT and planning CT images was quantitatively evaluated using commonly used metrics: mean absolute error (MAE), peak signal-to-noise ratio (PSNR), and the structural similarity measure index (SSIM). MAE provides an overall difference by comparing the voxel-wise value between the sCT and planning CT, and its value decreases as the image gets closer to the real one. PSNR is an indicator that measures the amount of noise in the sCT relative to the planning CT signal, with a higher value indicating better image quality. SSIM compares luminance, contrast, and structure between planning CT and sCT images. It produces a value between 0 and 1, with values closer to 1 indicating better similarity and image quality. Additionally, the histogram of intensity for CT and sCT images were compared using the Kullback-Leibler (KL) divergence (41). KL divergence measures the difference between two distributions, quantifying how much one distribution diverges from the other. A lower KL divergence implies that the those have similar distribution. Image metrics were calculated only within the external mask of the planning CT mask. Wilcoxon rank-sum test was used for statistical analysis (42). To qualitatively evaluate the sCT images, two certified radiation oncologists from authors’ institution rated the images using a 5-grade scale.

#### 2.4.2 Dosimetric evaluation of synthetic CTs

For each of the 20 test patients, dose calculations on sCT were performed using the same clinical plan applied to the planning CT, utilizing the Monte Carlo algorithm in the treatment planning system MONACO 5.51.11 (Elekta, Stockholm, Sweden) for Unity. The dose grid resolution was 2.0 × 2.0 × 2.0 mm^3^, and the statistical uncertainty per calculation was 1%. For dosimetric evaluation, gamma analysis was conducted between the dose distributions based on planning CT and sCT (43). The delivery quality assurance (DQA) criteria of authors’ institution was local gamma analysis with a 3%/3 mm, 10% dose threshold. Additionally, we conducted 1%/1 mm, 2%/2 mm, and 3%/3 mm gamma analysis for further evaluation, with the same dose threshold. To investigate the impact of anatomical differences between CT and MR on dose distribution, the 20 cases were divided into two groups: 10 for Group 1, where MR and CT showed good anatomical alignment, and 10 for Group 2, with less anatomical alignment. Subsequently, gamma analysis was conducted for each group, followed by a comparison of intensity distributions utilizing KL divergence. Additionally, for 10 patients with same organ-at-risks (OARs), planning target volume (PTV) and gross tumor volume (GTV) the average dose volume histogram (DVH) differences for each structure were calculated and evaluated. The OARs, PTV and GTV delineated on the planning CT by a certified radiation oncologist were rigidly copied to the sCT for assessment.

#### 2.4.3 Ablation study

Ablative study was performed for structure-conserving loss and generator. The comparisons were made between the baseline (original cycle-GAN), Swin only (modify cycle-GAN generator as Swin-UNETR), MIND only (cycle-GAN with MIND loss), and the proposed method (modify generator and use MIND loss).

## 3 Results

### 3.1 Image quality

**Figure 4:**
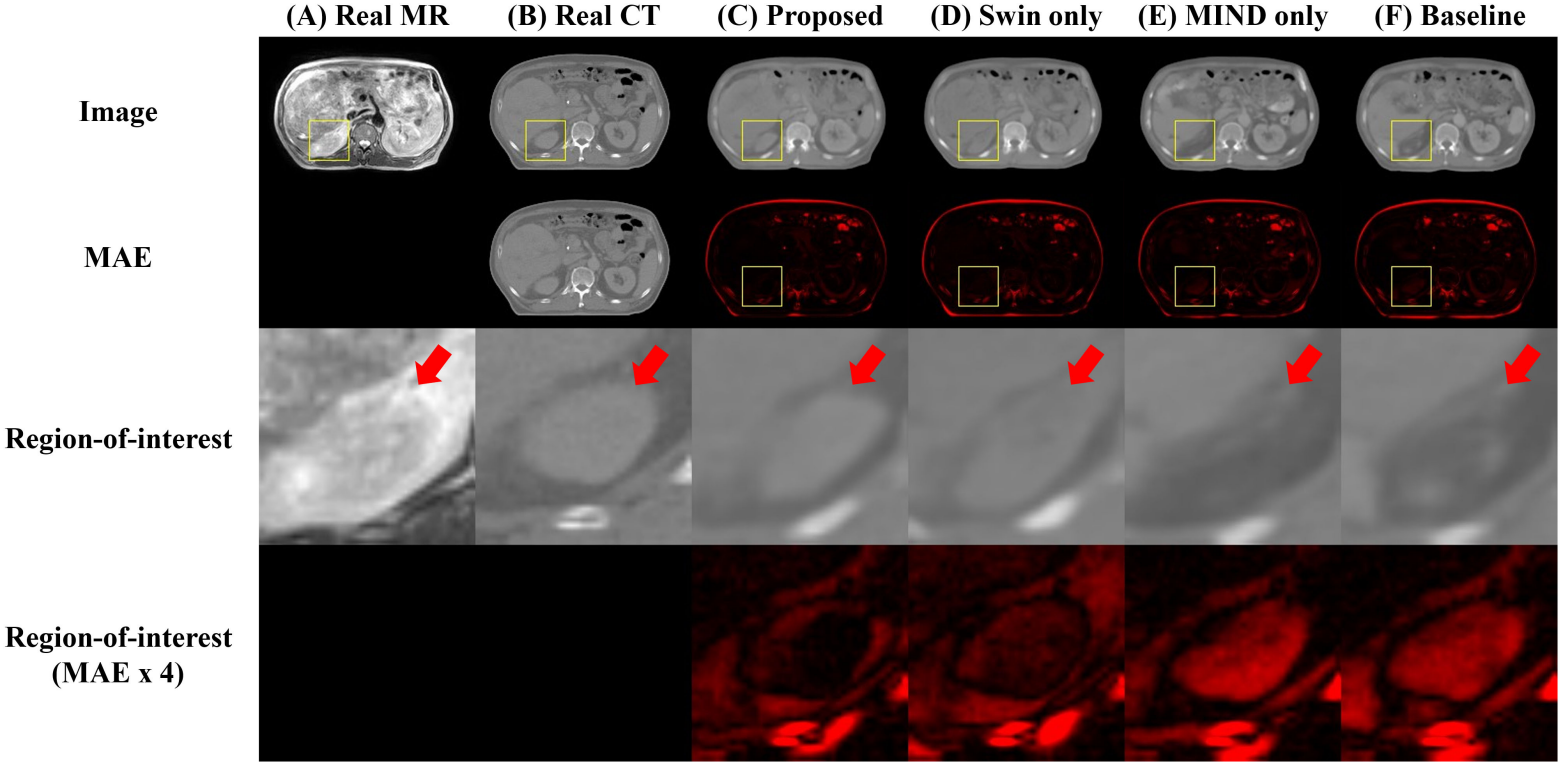
Comparison of computed tomography (CT) images as follows: **(A)** real MR, **(B)** real planning CT, sCT images generated by **(C)** proposed method, **(D)** Swin only, **(E)** MIND only, and **(F)** baseline models. The first and second row depict images and mean absolute error (MAE) with planning CT. The third and fourth row illustrate magnified right kidney (indicated by yellow square box) and MAE with planning CT. Red arrows highlighted the anatomical differences.

**Figure 5:**
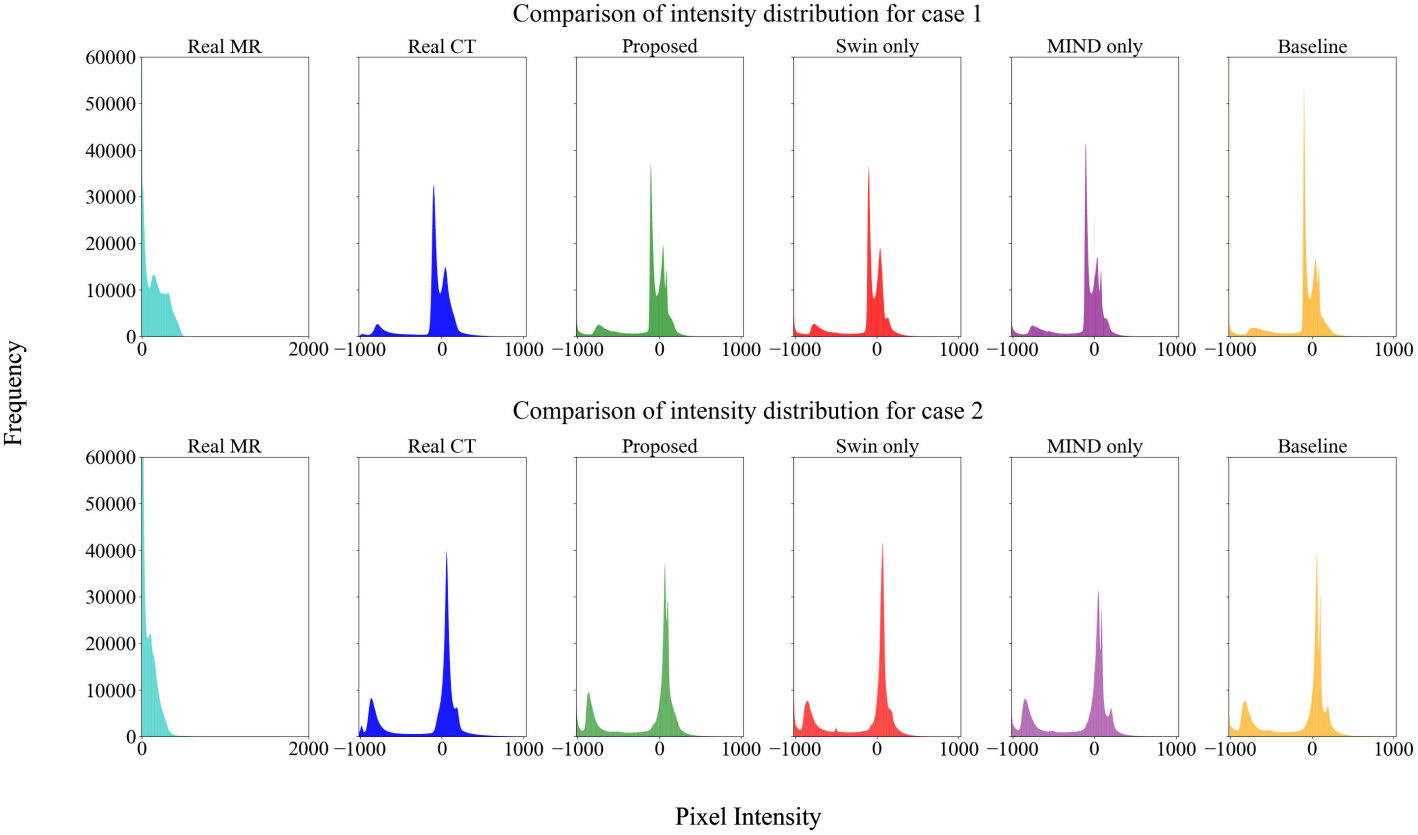
Histogram analysis of MR, CT, and sCT generated by each method. The sCT histogram generally aligns with the CT histogram, differing from the MR, indicating that style transformation has been effectively achieved.

**Figure 4** compared scanned MR, scanned CT, and generated sCT images of various methods. The proposed model produced sCT images with greater geometric consistency relative to the MR image and improved texture homogeneity with the planning CT image, especially in regions such as the kidney. The MAE, PSNR, and SSIM of proposed method were highest than other methods. **Table 2** indicates that applying Swin-UNETR and MIND loss individually did not result in statistically significant differences compared to the baseline. However, the combination of both methods led to statistically significant improvements in MAE and PSNR. The SSIM values were better with the proposed method, although the differences were not statistically significant. The KL divergence between the intensity histograms of CT and sCT demonstrated statistically significant differences from the baseline sCT across all cases: when using the proposed method, applying only Swin-UNETR, and applying only MIND loss. **Figure 5** depicted the histograms of MR, CT, and generated sCT images from various methods. **Table 3** presents the results of the qualitative evaluation of each sCT, conducted by two certified radiation oncologists.

**Table 2:** Mean ± standard deviation of MAE, PSNR, SSIM and KL divergence for synthetic CT images compared to planning CT images.

	MAE (HU) ↓	PSNR (dB) ↑	SSIM↑	KL divergence ↓
Proposed	79.5 ± 11.7*	27.1 ± 0.1*	**0.845 ± 0.034**	0.230 ± 0.070*
Swin only	83.6 ± 9.7	26.4 ± 0.8	0.832 ± 0.031	0.225 ± 0.053*
MIND only	80.5 ± 11.3	26.9 ± 1.0	0.841 ± 0.033	0.339 ± 0.090*
Baseline	86.1 ± 11.5	26.4 ± 0.9	0.828 ± 0.034	0.448 ± 0.070

Asterisks denote the statistically significant difference of metrics between proposed and the baseline method.(p-value < 0.05; Wilcoxon rank sum test). Bold values indicate the best values.

**Table 3:** Results of the qualitative evaluation of sCTs generated by each method.

	Proposed	Swin-only	MIND-only	Baseline
Expert 1	2.85 ± 0.85	2.15 ± 0.85	2.50 ± 0.74	2.65 ± 0.79
Expert 2	3.30 ± 1.14	2.95 ± 1.07	3.25 ± 1.13	3.15 ± 1.11
Total	3.08 ± 1.03	2.55 ± 1.05	2.88 ± 1.03	2.90 ± 0.99

Certified radiation oncologists assessed the images using a grade scale ranging from 1 (very bad) to 5 (very good). The results are presented as the mean ± standard deviation of the scores.

### 3.2 Dose evaluation

Dosimetric evaluation was performed by comparing dose distribution of the planning CT and sCT generation methods. **Table 4** describes the results of local and global gamma analysis for 20 patients based on a 10% threshold, conducted at 1%/1 mm, 2%/2 mm, and 3%/3 mm criteria.

**Table 4:** Results of local and global gamma analysis for dose distribution obtained from proposed, Swin only, MIND only, and baseline sCT.

	Gamma passing rate	p-value
	Local gamma analysis (3%/3 mm)	
*Proposed*	0.974 ± 0.012	**-**
*Swin only*	0.970 ± 0.013	0.43
*MIND only*	0.976 ± 0.011	0.32
*Baseline*	0.975 ± 0.013	0.53
	Global gamma analysis (3%/3 mm)	
*Proposed*	0.989 ± 0.008	–
*Swin only*	0.988 ± 0.008	0.70
*MIND only*	0.991 ± 0.007	0.59
*Baseline*	0.990 ± 0.009	0.63
	Local gamma analysis (2%/2 mm)	
*Proposed*	0.923 ± 0.027	–
*Swin only*	0.915 ± 0.029	0.37
*MIND only*	0.928 ± 0.027	0.47
*Baseline*	0.925 ± 0.029	0.73
	Global gamma analysis (2%/2 mm)	
*Proposed*	0.971 ± 0.027	–
*Swin only*	0.967 ± 0.018	0.48
*MIND only*	0.974 ± 0.015	0.34
*Baseline*	0.972 ± 0.018	0.50
	Local gamma analysis (1%/1 mm)	
*Proposed*	0.727 ± 0.064	–
*Swin only*	0.716 ± 0.065	0.53
*MIND only*	0.739 ± 0.067	0.47
*Baseline*	0.731 ± 0.066	0.79
	Global gamma analysis (1%/1 mm)	
*Proposed*	0.861 ± 0.059	–
*Swin only*	0.850 ± 0.062	0.50
*MIND only*	0.870 ± 0.060	0.47
*Baseline*	0.864 ± 0.061	0.68

Best value highlighted in bold. The p-values between the proposed method and the other methods were listed in the table.

**Figure 6:**
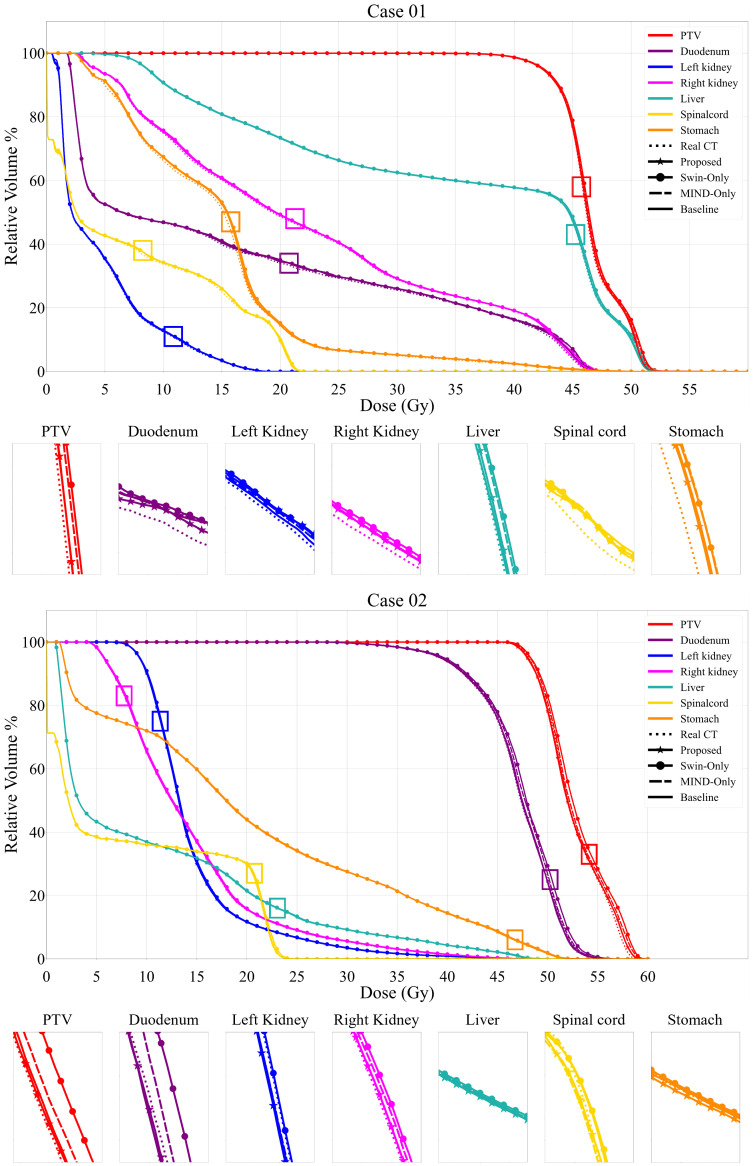
Comparison of dose volume histograms (DVH) for 2 cases, showing the comparison of the proposed method, Swin only, MIND only and baseline sCT and planning CT (solid lines) against the planning CT for the same OARs and each PTV. The PTV dosimetric criteria for Case 1 were V_4.75Gy_ > 95%. The V_4.75Gy_ in real CT was 99.20%, while the values in Proposed, Swin-only, MIND only, and Baseline were 99.25%, 99.43%, 99.38%, and 99.11%, respectively. In Case 2, with the same criteria, the V_4.75Gy_ in real CT was 98.91%, while the values in Proposed, Swin-only, MIND only, and Baseline were 98.09%, 99.11%, 98.82%, and 98.31%, respectively.

**Figure 7:**
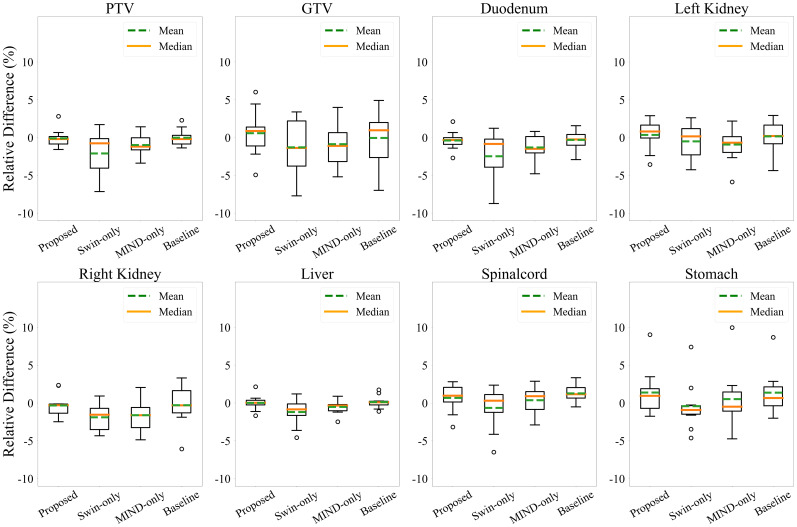
Relative dose volume histogram (DVH) differences for the PTV, GTV and organ-at-risks across the 10 patients. The DVH from proposed method had less than 5% average differences for all structures except the spinal cord and stomach.

**Figures 6** and **7** presents the relative DVH differences for the same OARs and GTV structures in 10 patients. The proposed method demonstrated a relative DVH difference within 5% compared to the planning CT, except for the spinal cord and stomach. This indicates that the dose distributions based on sCT from the proposed method closely matched those from the planning CT-based clinical plans, showing high consistency in dosimetric accuracy across various structures.

**Figure 8:**
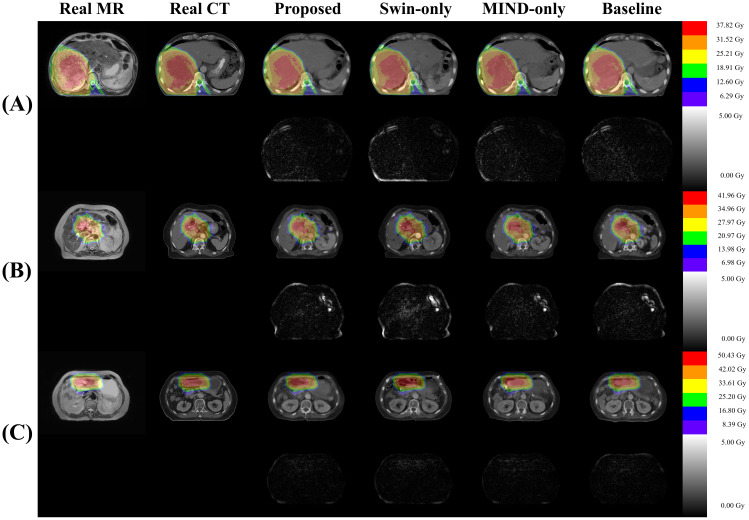
Comparison of dose distributions for magnetic resonance (MR), computed tomography (CT), and synthetic CT (sCT) images for three subjects **(A–C)**. Each subpanel includes an overlay of dose distribution on the images in first row and the dose differences in the second row.

**Figure 8** presents a comparison of dose distributions for (A) Real planning CT, (B) Proposed sCT, (C) Swin only sCT, (D) MIND only sCT, and (E) Baseline sCT methods. The figure shows the axial slices of the dose distribution with overlaid contour lines for critical structures and the target region.

**Figure 9:**
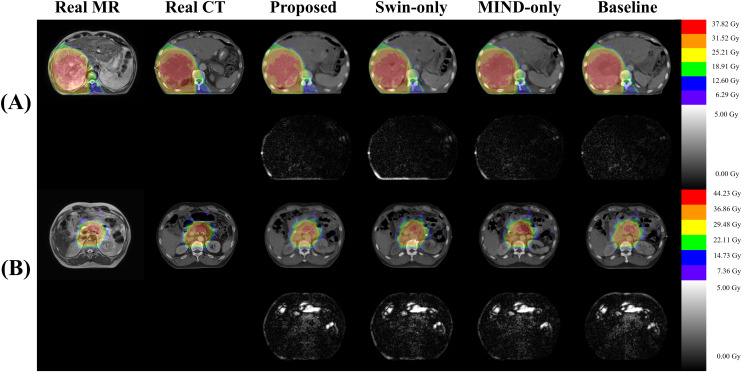
Visualization of dose differences according to anatomical structures. **(A)** shows a slice with relatively minor anatomical differences between CT and MR (Group 1), while **(B)** depicts a slice with more significant differences (Group 2). In **(B)**, distinct dose differences can be observed between the real CT and sCT due to differences in gas structures.

**Table 5** illustrates the impact of anatomical differences on dose distribution. In local gamma analysis, no statistically significant differences were found between the two groups at the 3%/3 mm and 2%/2 mm criteria. However, in global gamma analysis, statistically significant differences were observed at the 3%/3 mm and 2%/2 mm levels, except for the baseline sCT. **Figure 9** provides a qualitative comparison of dose distribution differences related to anatomical variation. In contrast, as illustrated in **Table 6**, there was no statistically significant difference between the two groups in the comparison of KL divergence results.

**Table 5:** Gamma passing rates for Group 1 (CT and MR with good anatomical alignment) and Group 2 (CT and MR with poor anatomical alignment), with p-values indicating differences between the groups.

	Group 1 Gamma passing rate	Group 2 Gamma passing rate	p-value
	Local gamma analysis (3%/3 mm)		
*Proposed*	0.976 ± 0.011	0.971 ± 0.012	0.29
*Swin only*	0.973 ± 0.012	0.967 ± 0.013	0.29
*MIND only*	0.978 ± 0.011	0.974 ± 0.010	0.26
*Baseline*	0.978 ± 0.012	0.972 ± 0.013	0.26
	Global gamma analysis (3%/3 mm)		
*Proposed*	**0.993 ± 0.006**	0.986 ± 0.008	**0.04**
*Swin only*	**0.992 ± 0.006**	0.984 ± 0.008	**0.03**
*MIND only*	**0.994 ± 0.006**	0.988 ± 0.007	**0.04**
*Baseline*	**0.994 ± 0.006**	0.987 ± 0.008	**< 0.05**
	Local gamma analysis (2%/2 mm)		
*Proposed*	0.924 ± 0.028	0.921 ± 0.026	0.55
*Swin only*	0.918 ± 0.029	0.913 ± 0.029	0.50
*MIND only*	0.929 ± 0.027	0.927 ± 0.026	0.65
*Baseline*	0.927 ± 0.029	0.922 ± 0.029	0.45
	Global gamma analysis (2%/2 mm)		
*Proposed*	**0.978 ± 0.013**	0.963 ± 0.017	**0.04**
*Swin only*	**0.975 ± 0.014**	0.959 ± 0.018	**< 0.05**
*MIND only*	**0.981 ± 0.012**	0.967 ± 0.015	**< 0.05**
*Baseline*	0.980 ± 0.013	0.964 ± 0.018	0.07

Statistically significant results are highlighted in bold.

**Table 6:** KL divergence for Group 1 (CT and MR with good anatomical alignment) and Group 2 (CT and MR with poor anatomical alignment), with p-values indicating differences between the groups.

	Group 1 KL divergence	Group 2 KL divergence	p-value
*Proposed*	0.231 ± 0.089	0.229 ± 0.044	0.33
Swin only	0.225 ± 0.067	0.225 ± 0.034	0.13
MIND only	0.343 ± 0.093	0.335 ± 0.086	0.82
Baseline	0.430 ± 0.058	0.466 ± 0.075	0.23

## 4 Discussion

**Figure 10:**
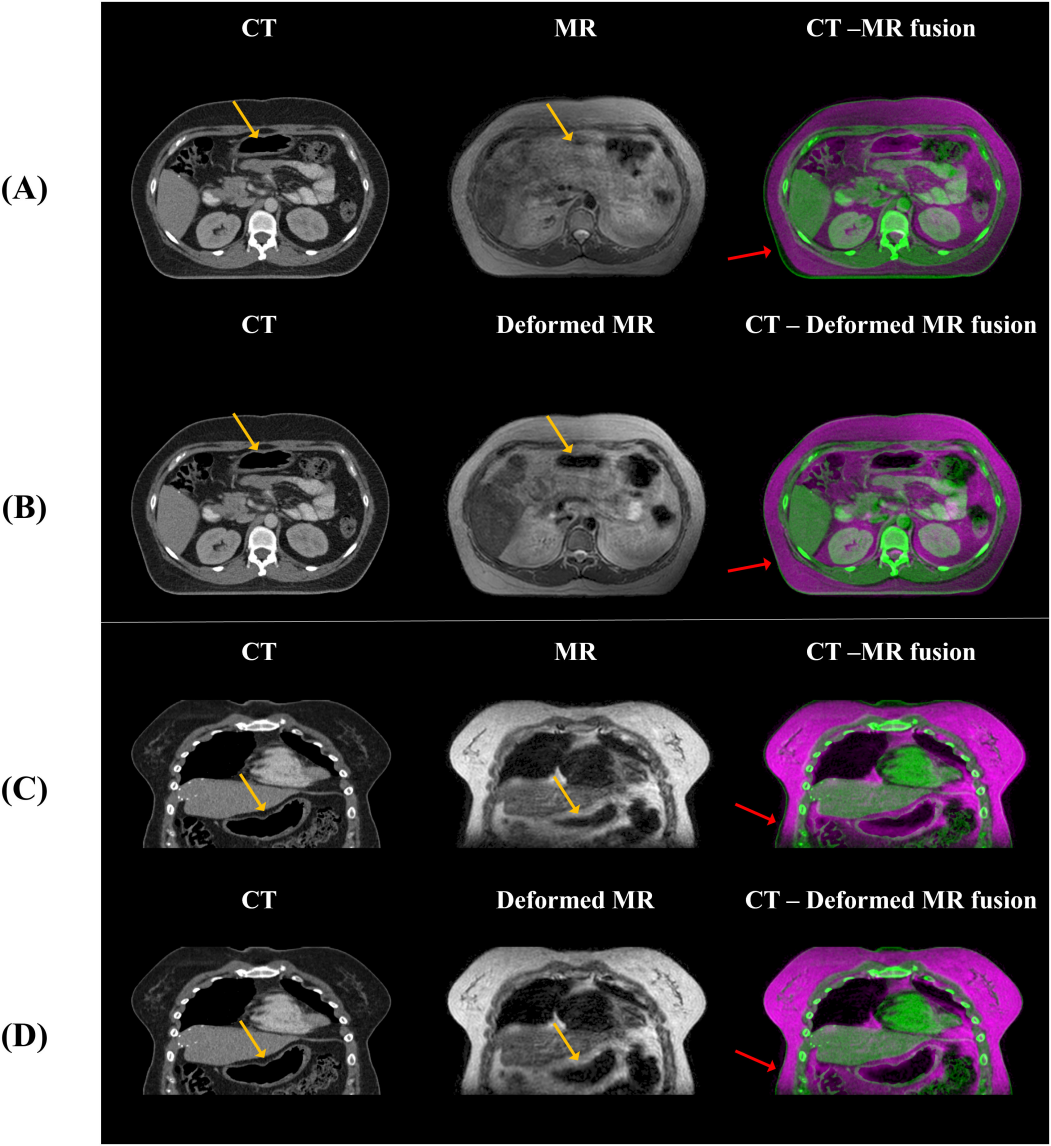
The anatomical differences between CT and MR, and between CT and deformed MR, are shown. **(A, B)** display axial views, while **(C, D)** show coronal views, illustrating that anatomical differences with CT are reduced when deformation is applied to the MR compared to the original MR. The yellow arrows indicate alignment of internal gas structures between CT and MR after deformable registration, while the red arrows show approximate alignment of the body external contour between CT and MR. However, the blue arrows highlight regions where intestinal gas structures still do not align between CT and MR.

In this study, we developed sCT generation from MR images of abdominal cancer patients using the unpaired data from 1.5 T MR-Linac. The primary task is image translation process that transforms style while preserving the content of MR images. However, image synthesis in abdominal region is often challenging than other body parts due to anatomical changes such as peristalsis and intestinal gas. To reduce anatomical difference, minimizing the time interval between the CT and MR scans are crucial. However, this study utilized retrospective data, and due to the predefined clinical protocol, it is procedurally challenging to acquire additional data beyond this framework. As shown in **Figure 10**, we conducted deformable registration as a preliminary step to mitigate anatomical differences between CT and MR.

This study focuses on stabilizing GAN training, addressing challenges of training instability and optimization difficulties in both the generator and discriminator (44). To achieve this, we employed the Adam optimizer with a learning rate of 0.0001, maintaining for the first 30 epochs. Subsequently, the learning rate was linearly decayed over the remaining 70 epochs. These configurations were designed to ensure smoother convergence during training. Additionally, patient data varied considerably in shape, resolution, and setup which could adversely affect the stability of cycle-GAN. To address this, we aligned the back positions of all patients based on the head-first supine position. Additionally, deformable registration was performed to enhance geometric correspondence between MR and planning CT images, as demonstrated in **Figure 10**. These preprocessing steps were crucial in improving the stability and reliability of our GAN-based approach, allowing network to focus on specific features while maintaining consistency in other aspects of the data.

Effective utilization of both global and local features was made using Swin-UNETR as a generator. Swin-UNETR combines the transformer structure of UNETR, which integrates a transformer into the traditional U-Net, with the Swin transformer (37, 38, 45). The Swin transformer extract both global and local features by leveraging both global and local attention (32, 45). Especially, as shown in **Figure 4**, applying Swin-UNETR as a generator maintained MR content in sCT images. Additionally, in the histogram analysis of MR, planning CT, and sCTs generated by each method in **Figure 5**, the proposed method qualitatively demonstrates successful style transformation between MR and planning CT.

Additionally, we employed MIND loss to further preserve structure since Kieselmann et al. (46) reported that cycle-GAN alone can result in subtle differences between MR and sCT. This is particularly important for this study since abdominal MR and CT often exhibit anatomical discrepancies due to factors such as imaging modality and peristalsis. While deformable registration can partially alleviate, it was challenging to mitigate them entirely (47). Therefore, it is necessary to utilize constraints on the geometric information between the input and output. MIND utilizes features extracted independently from MR and sCT intensities as in **Figure 3**, allowing direct structural comparison between MR and sCT (36). This method was previously applied in generating head and neck synthetic CTs using cycle-GAN and demonstrated better performance compared to when MIND loss was not applied (40). The proposed method showed a slight improvement in gamma analysis and DVH difference. Specifically, **Figure 7** indicates that the use of a Transformer structure helped in mitigating outliers, enhancing the overall robustness of the generated sCT images, leading to statistically significant improvements in image quality.

There are several related studies of sCT for MR-only radiotherapy. Cusumano et al. (28) utilized a conditional GAN (cGAN) on 20 test patients. They evaluated the image metrics using MAE and mean error (ME), achieving an MAE of 78.71 HU in the abdominal body region and an ME of 10.83 HU. In the dose evaluation, they achieved a gamma passing rate of 99% under the 2%/2 mm criteria and the mean dose difference in the PTV was -0.28%. Fu et al. (48) employed both cGAN and cycle-GAN networks, validating their results using leave-one-out cross-validation on 12 abdominal tumor patients. They evaluated the image metrics using MAE and PSNR, with the cGAN achieving an MAE of 89.8 HU and a PSNR of 27.4 dB, while the cycle-GAN achieved an MAE of 94.1 HU and a PSNR of 27.2 dB. In the dose evaluation, both cGAN and cycle-GAN achieved a gamma passing rate of 99% under the 3%/3 mm criteria and the mean dose difference in the PTV was -0.17%. The MAE values of proposed method (79.5 ± 11.7 HU) were comparable to those in Cusumano et al. (28) and slightly higher than the 89.8 ± 18.7 HU reported by Fu et al. (48). However, the gamma passing rates were not as high as the 99.8 ± 0.2% reported by Cusumano et al. or the 99.5 ± 0.7% Fu et al. (28) (48)There are several possible reasons for the differences. First, the regional difference between MAE and dose calculation. While the MAE was calculated within the patient body, dose calculation was conducted in relatively smaller regions than MAE due to dose thresholding. Second, MR imaging sequence. The previous studies predominantly utilized breath-hold MR images (28, 30, 48). In contrast, this study employed free-breathing MR images without breath-hold which includes more artifacts. Despite of this, our results demonstrate a comparable quantitative results. Lastly, local gamma pass ratio. This study utilized local gamma analysis, providing a stricter assessment of geometric alignment and intensity consistency compared to the conventional global gamma analysis (49).

In the case of DVH difference, 5% is generally considered as the action level according to the TG-119 report and TG-218 report (50, 51). However, to the best of the authors’ knowledge, no specific guidelines for acceptable differences between calculation methods are clearly defined. Therefore, in this study, the dose criteria were referenced for different treatment plans for each patient, and **Figure 6** shows that the synthetic CT satisfies the dose criteria used in the planning CT. This judgement is informed by radiation oncologists’ and physicists’ expertise, clinical experience, and the specific anatomical and functional considerations relevant to each case. Thus, the observed differences in DVH values are interpreted within the context of these patient-specific clinical priorities, allowing for variability in assessment depending on the unique circumstances of each treatment plan. Furthermore, as illustrated in **Figure 7**, the proposed method demonstrated robust performance, with differences within 5% for PTV and OAR overall except stomach and GTV. The mean dose differences were -0.09 ± 1.16%, -2.10 ± 2.96%, -0.98 ± 1.40%, and -0.02 ± 1.10 for Proposed, Swin-only, MIND-only, and baseline, respectively, in the PTV; -0.38 ± 1.20%, -2.46 ± 3.37%, -1.31 ± 1.68%, and -0.30 ± 1.28% in the GTV; and 0.58 ± 2.99%, -1.28 ± 3.59%, -0.87 ± 2.95%, and -0.04 ± 3.44% in the duodenum. One case in the stomach showed an outlier for all methods. This was attributed to the limitations of the unpaired dataset, where a high signal in the CT intestine resulted in a dose discrepancy in the stomach.

In this study, we referred to the manufacturer’s recommendations for acceptable gamma passing rates due to the lack of specific criteria for gamma passing rates among calculations. They recommend a gamma criterion of 3%/3mm with a 10% threshold and global gamma analysis for delivery quality assurance, noting that a passing rate above 95% is considered acceptable under these criteria (52). Under the 3%/3mm criterion with global gamma analysis, we achieved a gamma passing rate of 99%, exceeding the 95% threshold typically used for comparing calculations and measurements. In our study, the gamma passing rates for global gamma analysis were as follows: 86.1 ± 5.9% for 1%/1mm, 97.1 ± 2.7% for 2%/2mm, and 98.9 ± 0.8% for 3%/3mm. These results showed consistency with previous studies, even though our studies employed free-breathing abdominal MR which have more artifacts. For example, Olberg et al. (30) reported a gamma passing rate of 98.3% ± 1.3% for the 3%/3mm criterion. Similarly, Fu et al. (48) demonstrated passing rates of 98.5% ± 2.8% for 2%/2mm and 99.5% ± 0.7% for 3%/3mm. Cusumano et al. (28) reported gamma passing rates of 90.8% ± 4.5%, 98.7% ± 1.1%, and 99.8% ± 0.2% for 1%/1mm, 2%/2mm, and 3%/3mm, respectively. However, they did not specify their gamma analysis global or local.

Although proposed method successfully generated sCT, there are few limitations. First, uncertainties of evaluation between sCT and CT. Since MR images are not scanned immediately after the planning CT, there are inherent geometric discrepancies between the sCT generated from online MR and the planning CT (22). Those anatomical discrepancies including intestinal gas and weight change result in inaccurate evaluation of image and dosimetric evaluations. **Figure 9** and **Table 5** demonstrates the dose differences attributable to anatomical discrepancies between MR and CT, but **Table 6**, which compares the intensity distributions of the images, shows no statistically significant differences between groups 1 and 2. Second, artifacts in the MR images limited the quality of the sCT. The MR data used in this study were online MR images captured during abdominal treatment with Unity. However, artifacts caused by respiratory and organ movements were still present, and the kidneys were not well distinguished from surrounding organs in the MR images. These limitations in the input MR conditions resulted in a decrease in the overall quality of the sCT. These artifacts could be mitigated by breathing considered sequences (e.g., shallow breathing, breath holding) or using software-based corrections (53–56). Lastly, while the proposed method’s sCT showed the best results in terms of image metrics and qualitative comparison, there were no statistically significant differences in gamma passing rates compared to the other methods. Although correlation between the MAE and the gamma passing rate was reported, unpaired dataset and different calculation region between MAE and gamma pass ratio due to the dose thresholding, the correlation between gamma pass rate and MAE could be weaker (57). Similarly, in the comparison of cGAN and cycle-GAN conducted by Fu et al. (48), the MAE values were 89.8 ± 18.7 HU and 94.1 ± 30.0 HU, respectively, while the 3%/3 mm gamma analysis with a 30% dose threshold showed passing rates of 99.5 ± 0.8% and 99.5 ± 0.7%, respectively.

## 5 Conclusion

This study proposed the generation of sCT images from MR images obtained from 1.5T MR-Linacs using cycle-GAN. By modifying the generator to Swin-UNETR and incorporating a structure-conserving loss, proposed method was able to enhance both image quality and dosimetric accuracy for abdominal cancer patients. An ablation study demonstrated that the proposed method improves geometric consistency and texture homogeneity of sCT images compared to other models. This study underscores the importance of considering both local and global features, and structure preservation for sCT.

## Data Availability

The raw data supporting the conclusions of this article will be made available by the authors, without undue reservation.
